# Role of the Insect Neuroendocrine System in the Response to Cold Stress

**DOI:** 10.3389/fphys.2020.00376

**Published:** 2020-04-23

**Authors:** Jan Lubawy, Arkadiusz Urbański, Hervé Colinet, Hans-Joachim Pflüger, Paweł Marciniak

**Affiliations:** ^1^Department of Animal Physiology and Development, Faculty of Biology, Institute of Experimental Biology, Adam Mickiewicz University Poznań, Poznań, Poland; ^2^HiProMine S.A., Robakowo, Poland; ^3^ECOBIO – UMR 6553, Université de Rennes 1, CNRS, Rennes, France; ^4^Neurobiology, Institute of Biology, Freie Universität Berlin, Berlin, Germany

**Keywords:** cold stress, neurohormones, insects, biogenic amines, neuroendocrinology, neuropeptides

## Abstract

Insects are the largest group of animals. They are capable of surviving in virtually all environments from arid deserts to the freezing permafrost of polar regions. This success is due to their great capacity to tolerate a range of environmental stresses, such as low temperature. Cold/freezing stress affects many physiological processes in insects, causing changes in main metabolic pathways, cellular dehydration, loss of neuromuscular function, and imbalance in water and ion homeostasis. The neuroendocrine system and its related signaling mediators, such as neuropeptides and biogenic amines, play central roles in the regulation of the various physiological and behavioral processes of insects and hence can also potentially impact thermal tolerance. In response to cold stress, various chemical signals are released either via direct intercellular contact or systemically. These are signals which regulate osmoregulation – capability peptides (CAPA), inotocin (ITC)-like peptides, ion transport peptide (ITP), diuretic hormones and calcitonin (CAL), substances related to the general response to various stress factors – tachykinin-related peptides (TRPs) or peptides responsible for the mobilization of body reserves. All these processes are potentially important in cold tolerance mechanisms. This review summarizes the current knowledge on the involvement of the neuroendocrine system in the cold stress response and the possible contributions of various signaling molecules in this process.

## Introduction

Insects are the largest group within the arthropod phylum. They are capable of surviving in virtually every environment from the deserts of Africa through the grasslands of temperate zones to the freezing permafrosts of Arctic regions (Chown and Nicolson, 2004). A major factor determining insect species distributions is their cold tolerance and water availability (Addo-Bediako et al., 2000). During their evolution, insects subjected to low temperature have developed distinct adaptations to overcome and thrive in suboptimal thermal conditions (Wharton, 2007; Lee, 2010). To survive in environments where the temperature drops below freezing, insects have evolved diverse mechanisms, which can be divided into two main strategies: (i) freeze-tolerance and (ii) freeze-avoidance (Lee, 1991; Sømme, 1999; Sinclair et al., 2003). In freeze-tolerant species, freezing is limited only to extracellular matrix (ECM), as the formation of ice crystals inside of the cell inevitably leads to death of most animals (Storey and Storey, 1989; Block, 2003). The second strategy is much more widespread among arthropod phyla (Block, 1990; Lee and Costanzo, 1998), and freeze-avoiding insects utilize mechanisms which raise their ability to stay unfrozen by supercooling (Sformo et al., 2010). Cold and freezing stress affects a large number of physiological processes (Teets and Denlinger, 2013), causing mechanical damage to cells or their DNA (Lubawy et al., 2019), changes in main metabolic pathways (Chowanski et al., 2015, 2017b) or cellular dehydration, which results in increased acidity, toxic metabolic intermediate concentrations and osmotic stress (Storey and Storey, 2012; Pegg, 2015; Des Marteaux and Sinclair, 2016; Andersen et al., 2018). In both of these strategies, cryoprotectants are necessary for survival. Cryoprotectants like glycerol, trehalose or glucose, which lower the lowest lethal temperature are synthetized by freeze-avoiding species. The increase in their concentration results also in a drop of supercooling point (SCP) (Zachariassen, 1985). The freeze-tolerant insects in turn utilize these molecules to reduce cellular dehydration since ice formed in the ECM attracts water out of cells (Storey and Storey, 1988). Species that cannot tolerate freezing also remove any particles that can start the ice nucleation process, such as food, dust or bacteria from gut or ECM. This may be achieved for example by inhibiting feeding (Olsen and Duman, 1997). The latest findings indicate that water and ion balance is crucial for withstanding chilling injuries that lead to chill coma and death (Overgaard and MacMillan, 2017). Therefore, nerves and muscles are highly susceptible to cold stress (Garcia and Teets, 2019). However, little is known about the role of the nervous system in orchestrating these finely tuned processes.

The nervous and endocrine systems, through the process called neuroendocrine integration, interplay together to regulate a number of physiological functions and maintain system-wide homeostasis in regular as well as stressful situations (Hartenstein, 2006; Adamski et al., 2019). A number of physiological processes are mediated by two main classes of neurosecretory molecules i.e., neuropeptides and biogenic amines (Hartenstein, 2006; Chowanski et al., 2016, 2017a). They are produced mainly in the central nervous system (CNS) and take part in the regulation of metabolism, ion homeostasis and muscle contractions, including the heartbeat (Chowanski et al., 2017c). In different insect species, neuropeptides with homologous structures very often have similar functions (Bendena, 2010). As these compounds play central roles in physiological and behavioral processes, directly affecting the survival of adverse environmental conditions, it can be expected that in response to cold stress, molecules responsible for osmoregulation, such as capability peptides (CAPA), inotocin (ITC), ion transport peptide (ITP), diuretic hormones (DH_31_ and DH_44_), kinins and calcitonin (CAL), will take part. Substances related to the general response of insect organism to stressors such as tachykinin-related peptides (TRPs) and/or peptides responsible for the mobilization of reserve substances (e.g., glycogen, trehalose, and glucose) such as adipokinetic hormones (AKHs), insulin-like peptides (ILPs), or neuropeptide F (NPF) may also take part in the cold stress response, as they regulate metabolic homeostasis, the circadian clock and feeding (Gäde, 2004, 2009; Fadda et al., 2019). However, not only neuropeptides can be key players. Current knowledge shows that biogenic amines such as octopamine (OA), dopamine (DA), and serotonin (5-HT) are involved in the stress response (Gruntenko et al., 2004, 2016). The levels of the above-mentioned biogenic amines have been found to change in various insect species under unfavorable conditions, including high- or low-temperature stress (Hirashima et al., 2000; Chentsova et al., 2002). Hence, this paper summarizes the existing knowledge on the role of the neuroendocrine system in response to cold stress and research perspectives in this area.

## Biogenic Amines

Biogenic amines play a crucial role in the regulation of basic life processes (Farooqui, 2012; Sinakevitch et al., 2018). They act not only as neurotransmitters and neuromodulators in nervous tissues but also, depending on the situation, they can be released into body fluids and act as neurohormones (Sinakevitch et al., 2018). Biogenic amines bind to G-Protein coupled receptors (GPCRs) and, depending on the receptor type and target tissue, stimulate different types of secondary messengers, mainly cAMP or Ca^2+^ (Farooqui, 2012).

The main biogenic amines identified in insects are octopamine (OA), serotonin (5-HT), dopamine (DA), histamine (HA), and tyramine (TA) (Blenau and Baumann, 2001). Current knowledge about the role of biogenic amines in insects suggests a wide spectrum of actions. They participate in the regulation of many behaviors, such as locomotion, feeding or social interactions (Blenau and Baumann, 2001; Armstrong and Robertson, 2006; Pflüger and Duch, 2011). Biogenic amines also evoke systemic responses to different environmental factors, including stressful conditions or pathogen infection (Gruntenko et al., 2004; Adamo, 2008). For instance, research has shown that OA and DA are released into insect hemolymph in the first minutes after exposure to stress, which evokes a cascade of reactions leading to the re-attainment of homeostasis (Hirashima and Eto, 1993; Chentsova et al., 2002; Gruntenko et al., 2004). Interestingly, the release of these biogenic amines during stress conditions appears non-specific to stressor type. For example, heat, vibration and starvation trigger the same response (Orchard et al., 1981; Hirashima et al., 2000; Gruntenko et al., 2004). Likewise, winter conditions such as low temperatures and a short-day photoperiod induce changes in the concentrations of biogenic amines, which allow insects to survive unfavorable conditions and/or prepare them for prolonged stress conditions (Isabel et al., 2001; Armstrong and Robertson, 2006). However, the changes in biogenic amine concentrations and their cause may be different in the case of the response of insects to rapid exposure to cold and during the acclimation process before winter.

One of the most important effects of biogenic amine release to the insect hemolymph is the mobilization of energy (Lorenz and Gäde, 2009). The mobilization of energy prepares insects to higher metabolic activity related to the stress response and is useful during the recovery period (Farooqui, 2012). The mobilization of energy under the control of biogenic amines is the result of the stimulation of glycogen conversion into trehalose, glucose and trehalose oxidation and the release of lipids from fat body (Gruntenko et al., 2004). This action of biogenic amines may be very important through the prism of response to short-term and prolonged cold. Elevated levels of biogenic amines also intensify the process of energy mobilization by stimulating the release of other hormones, especially neuropeptides, which participate in the regulation of insect metabolism. The cooperation between biogenic amines and neuropeptides may evoke a reaction cascade that is crucial for the response of insects to environmental stressors, including cold. Pannabecker and Orchard (1986) showed that OA stimulates the release of AKHs. AKHs are neuropeptides that are considered the main insect stress hormones because, similar to OA, they enhance available energy by inducing lipolysis and suppressing life processes that have relatively low priority during stress conditions (Gäde, 2009; Ibrahim et al., 2018). The close interplay between these two hormones highlights the fact that receptors for AKHs were also found in dorsal unpaired median neurons (DUMs), which are among the main components of the insect octopaminergic system (Wicher et al., 2006; Wicher, 2007). Another example of close relations between biogenic amines and neuropeptides is the fact that the activity of neurosecretory cells producing ILPs is mediated by the serotonin receptor 5-HT_1__*A*_ and octopamine receptor OAMB (Luo et al., 2012). The detailed relationship between biogenic amines and ILPs is described in the Neuropeptides section (subsection Metabolism).

Cold acclimation allows the maintenance of metabolic homeostasis and insect survival under prolonged stress (Lalouette et al., 2007; Colinet et al., 2012; Enriquez and Colinet, 2019). Generally, acclimation is associated with changes in insect metabolites, including sugars, polyols, free amino acids (FAAs), proteins and also biogenic amines (Isabel et al., 2001; Lalouette et al., 2007; Colinet et al., 2012). A study by Isabel et al. (2001) clearly showed that the concentration of DA in diapausing *Pieris brassicae* pupae was higher than that in non-diapausing individuals. Moreover, the DA level progressively increases during diapause. In the case of the 5-HT level, in the initial phase of the pupal stage, Isabel et al. (2001) did not observe any changes between diapausing and non-diapausing pupae. However, the 5-HT concentration in diapausing pupae was stable, while in non-diapausing individuals, it dropped during this developmental stage. High concentrations of DA likely lead to the arrest of insect development. High 5-HT levels could participate in the inhibition of pupal metabolism, which may be crucial for survival during insect overwintering at this developmental stage. Interestingly, the results of Isabel et al. (2001) suggest that in the case of *P. brassicae*, the accumulation of biogenic amines is the result of changes in the photoperiod but not exposure to lower temperature. On the other hand, research conducted on the beetle *Alphitobius diaperinus* indicates the influence of cold exposure on increasing concentrations of tyrosine (Tyr), a precursor of many hormones, including OA and DA, in insect hemolymph (Lalouette et al., 2007). A strict correlation between biogenic amines and Tyr was also shown in a study performed by Rauschenbach et al. (1995), who demonstrated that during an increase in DA concentration, a simultaneous decrease in Tyr was observed in *Drosophila*. Moreover, research conducted on *Drosophila virilis* showed that cold acclimation led to the upregulation of genes encoding serotonin receptor 7 and the serotonin transporter, which may also suggest that 5-HT is likely important in regulation of response to prolonged thermal stress (Vesala et al., 2012).

Current research addressing the role of biogenic amines in response to short-term and prolonged cold suggests that these hormones are not only important in the regulation of insect metabolism but also participate in neuroprotection. Generally, insects enter coma at critical high and low temperatures (Rodgers et al., 2010; Armstrong et al., 2012; Srithiphaphirom et al., 2019). This physiological state partly results from the progressive loss of ion homeostasis. The alteration of ion equilibrium provokes the depolarization of membranes, altering the action potentials of muscles and neuron cells, leading to a loss of neuromuscular functions and coma (Overgaard and MacMillan, 2017). The alteration of ion concentrations across membranes is associated with a decrease in Na^+^/K^+^-ATPase activity at low temperature (McMullen and Storey, 2008). Interestingly, after temperature acclimation in three cockroach species, *Periplaneta americana, Leucophaea maderae*, and *Blaberus craniifer*, their nervous tissues were excitable at temperatures lower than previously determined temperatures, which induced a chill coma in non-acclimated individuals (Anderson and Mutchmor, 1968). Similar results were observed in *Drosophila* species. The pre-exposure of flies to low temperatures decreases the value of the critical thermal minimum (CT_*min*_), the temperature at which individuals lose responsiveness (Overgaard et al., 2011; Andersen et al., 2018). Interestingly, in *Locusta migratoria* after the application of OA decreasing of CT_*min*_ was observed (Srithiphaphirom et al., 2019). These data suggest that OA may play some role(s) in modulating the responsiveness of the nervous system under thermal stress. The OA mode of action is most likely associated with the indirect modulation of Na^+^/K^+^-ATPase activity and compensation for the negative effect of low temperatures on this pump (Srithiphaphirom et al., 2019). The activation of the OA receptor (i.e., OAR3) leads to the stimulation of cAMP production and activation of cAMP-dependent protein kinase A (PKA), which may regulate K^+^ channels and Na^+^/K^+^-ATPase pumps (Feschenko et al., 2000; Armstrong and Robertson, 2006; Srithiphaphirom et al., 2019).

Many studies have been conducted on the neuroprotective role of biogenic amines in maintaining the muscle activity of different crustaceans under stress conditions, including low temperature (Stephens, 1985, 1990; Hamilton et al., 2007). Generally, an increase in the OA and 5-HT concentration in the hemolymph associated with exposure to cold causes an increase in excitatory postsynaptic potential (EPSP) amplitude in lobster and crayfish muscles. The effect of the application of biogenic amines is very often temperature dependent; for example, 5-HT induces changes in EPSP only at temperatures lower than optimal. This phenomenon may help neuromuscular junctions remain functional at low temperatures (Hamilton et al., 2007; Zhu and Cooper, 2018). Based on these results, similar dependencies may also be observed in insects. This supposition is partially confirmed by the results of Zhu et al. (2016) in a study on the *D. melanogaster* heart. The authors demonstrated a strong excitatory effect of biogenic amines on the larval heart during cold exposure but only in the case of 5-HT. Interestingly, at room temperature, all of the tested biogenic amines (OA, DA, and 5-HT) evoke positive chronotropic effects on the *Drosophila* heart. Moreover, high concentrations (10 μM) of OA and DA at a low temperature led to a decrease in heart rate frequency or heart cessation. This negative chronotropic effect was not observed in the cases of OA and DA at low concentrations (1 μM). However, the strict physiological role of this OA and DA action is not fully understood. However, as suggested by Zhu et al. (2016), different modes of action of OA at different temperatures may be related to the activation of different subunits of the G protein-coupled receptor. The Gαq subunit, whose activation evokes a positive chronotropic effect, is most likely suppressed, but Gαi-coupled receptors are activated, which may lead to the opposite effect of OA on insect heart during cold stress (Zhu et al., 2016). Generally, biogenic amines may be needed to maintain heart functioning during chronic exposure to cold. This is essential for circulating nutrients/cryoprotectants and immune function, which undoubtedly influence insect survival during exposure to cold (Zhu et al., 2016).

**FIGURE 1:**
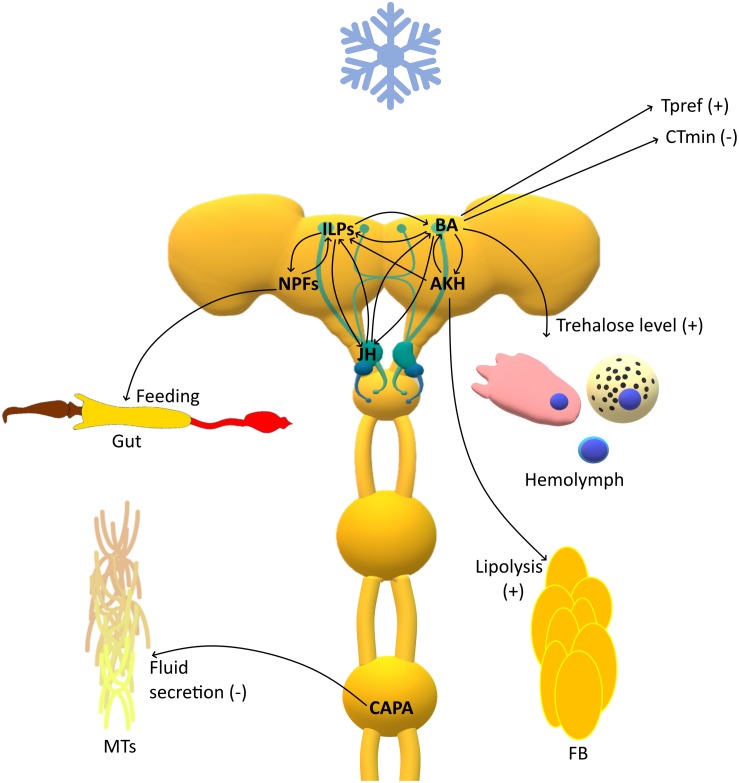
Schematic representation of the response of insect neuroendocrine system to cold stress. The role of certain compound can be multiple and tightly regulated by a number of feedback loops which actions add up to the effect of regulating these finely tuned processes. ILPs, Insulin like peptides; BA, Biogenic amines; JH, Juvenile hormone; NPFs, Neuropeptides F; AKH, Adipokinetic hormone; CAPA, capability peptide; MTs, Malpighian tubules; FB, Fat body; CT_*min*_, critical thermal minimum; T_*pref*_, preferred temperature; (+), increase; (−), decrease.

All insects have preferred temperature (Tpref) that maximize their metabolic activities and fitness (Crickenberger et al., 2019). Hence, they actively choose to occur in certain microenvironments to remain close to these temperatures (Dillon et al., 2009). In addition, in many situations, insects can avoid stressful conditions by moving into protected buffer microhabitats (Dillon et al., 2006). Recent research has shown that biogenic amines, especially DA and HA, participate in the regulation of Tpref in insects (Figure 1) (Hong et al., 2006; Bang et al., 2011; Tomchik, 2013). Bang et al. (2011) demonstrated that dopaminergic neurons located in mushroom bodies participate in the regulation of Tpref in *D. melanogaster*. The targeted inactivation of these neurons caused a loss of cold avoidance by flies. Moreover, mutation in the DA receptor gene led to a decrease in Tpref in *Drosophila* flies (Bang et al., 2011). Similar results were observed in DA transporter-defective mutants. Interestingly, in these mutants, a higher metabolic ratio was observed, which may suggest that differences in Tpref may be associated with disturbance to the equilibrium of heat gain and heat loss (Ueno et al., 2012). Additionally, the mutation of genes involved in HA signaling gave similar effects as previously mentioned for mutations in the DA system. Since HA participates in visual reception, these results indicate a putative relationship between temperature perception and the circadian clock, which may be crucial for the acclimation process (Hong et al., 2006).

Despite the regulatory actions of biogenic amines during the direct response to cold, these compounds protect against other environmental stressors, such as starvation (Krashes et al., 2009; Yang et al., 2015; Damrau et al., 2017). Moreover, biogenic amines inhibit energetically costly processes, such as reproduction, by stimulating JH degradation (Chentsova et al., 2002; Gruntenko et al., 2016). Starvation and reproductive arrest are processes that are particularly relevant to the cold tolerance of insects.

## Neuropeptides

### Diuresis

Below a certain low temperature, insects generally enter into chill coma, a state associated with neuromuscular paralysis (Mellanby, 1939; MacMillan and Sinclair, 2011b; Findsen et al., 2014). During this state, insects lose ion and water homeostasis and regain it during a process called chill coma recovery (CCR) (MacMillan et al., 2012). In insects, Malpighian tubules (MTs) and the gut are mainly responsible for the regulation of ion homeostasis. This process may vary quite noticeably between different insects, depending, for example on diet (O’Donnell, 2008). Typically, the MTs are responsible for production of the primary urine, which is more or less isosmotic with the hemolymph. Ions K^+^ and Cl^–^ (and Na^+^ in blood feeding insects) flow from hemolymph to the lumen due to the coupling of the V-ATPase and H^+^-cation exchangers. This allows to maintain a water gradient mediated by aquaporins, and movement of waste products by specific transporters into the lumen of the MTs (Ramsay, 1954; O’Donnell, 2009; Spring et al., 2009). The main neuropeptides contributing to the functioning of MTs are CAPAs, which stimulate or inhibit secretion depending on the insect species and life stage (Davies et al., 2013; Halberg et al., 2015); kinins, which, in addition to stimulating secretion in MTs, also control the activity of gut muscles (Coast et al., 1990; Dow, 2009); and diuretic hormones (DH_31_ and DH_44_) (Te Brugge et al., 2011; Cannell et al., 2016). In insects, hormones causing a reduction in diuresis have been identified, including ITP (Audsley et al., 1992; Gáliková et al., 2018), neuroparsins, glycoproteins GPA2/GPB5 which possibly act as Cl^–^ transport stimulating hormone (CTSH) (Paluzzi, 2012; Paluzzi et al., 2014; Rocco and Paluzzi, 2016), CCHamide which affect both MTs and midgut (Capriotti et al., 2019) and antidiuretic factors a and b (ADFa and ADFb) (Eigenheer et al., 2002, 2003; Massaro et al., 2004). As cold and desiccation both may result in a reduction in hemolymph volume and an increase in osmolarity and are closely linked at the molecular level (Sinclair et al., 2007, 2013; Rajpurohit et al., 2013), these two stressors should always be considered together. Terhzaz et al. (2015) showed that in drosophilids, the non-lethal exposure to low temperature significantly increases the mRNA levels of *capa*. The increase in *capa* expression was dependent on the duration of stress and came back to the levels before stress, after 4 h of recovery. During recovery, CAPA neuropeptides are released from neuroendocrine cells, improving (reducing) CCR (Terhzaz et al., 2015). On the other hand, recently published study by Li et al. (2020) showed significant decrease in *capa* expression level after 4 h of cold stress (4°C) and no changes after 1 h, in *Bemisia tabaci*. Together with the changes in neuropeptide precursor level, decrease in expression level of CAPA receptor was also observed (Li et al., 2020). The Terhzez’s group also showed that during recovery from cold stress, the mRNA level of leucokinins increases in *Drosophila suzukii* (Terhzaz et al., 2018). Similar effect was also observed in *B. tabaci.* After prolonged cold stress (4°C for 4 h) a tendency to an increase in LK expression was noted (Li et al., 2020). These peptides also affect the function of MTs in *Aedes aegypti*, depolarizing them and increasing fluid secretion (Veenstra et al., 1997). This in turn shows that kinins may also take part in the response to cold stress (Terhzaz et al., 2018). Alford et al. (2019a) tested the effects of biostable analogs of kinin, CAPA, and PK in *D. suzukii* and *D. melanogaster*. They studied five CAPA/PK and three kinin analogs *ex vivo* to elucidate their roles in the modulation of fluid secretion through the MTs and *in vivo* to evaluate impacts of these neuropeptides on starvation, desiccation and cold stress tolerance. Out of all the tested peptides, the kinin analogs increased the fluid secretion in the MTs of both flies, whereas none of the other analogs affected this process. Although they did not affect the secretion of fluids, CAPA/PK analogs could be important regulators of stress response under desiccation conditions. Indeed, the injection of CAPA/PKs analogs increased survival under desiccation stress (Alford et al., 2019a). At low temperatures, injections of these analogs caused the protective effect, but only in *D. melanogaster* males and not in females of this species or in both sexes of *D. suzukii* (Alford et al., 2019a). In another study this group showed that 9 out of 10 tested analogs increased the mortality of cold stressed aphid *Myzus persicae* (Alford et al., 2019b). It has to be noted that in aphids MTs are not present due to evolutionary loss of these organs, and the osmoregulatory function of these organs was taken over by the gut (Jing et al., 2015). MacMillan et al. (2018) also showed that CAPA was connected to the cold tolerance of *D. melanogaster*, although the effects were dose-dependent (Figure 1). When administered at very low, femtomolar concentrations, CAPA was anti-diuretic and reduced tubule K^+^ clearance rates and chill tolerance by significantly increasing the CCR time. However, at high doses, it facilitated K^+^ clearance from the hemolymph and increased chill tolerance by reducing the CCR time and increasing survival (MacMillan et al., 2018).

As mentioned before, DH_31_ and DH_44_ represent another potential candidates for the investigation of the role of the neuroendocrine system in the cold stress response. However, the number of studies on this topic is limited. Although the results found by Terhzaz et al. (2018) showed no change in the mRNA level of DH_31_ and DH_44_ after cold stress, one should consider the possibility that results solely obtained from a single strain of *Drosophila* spp. may not be representative of the whole family/genus, as there is significant genetic variability in stress response among various populations of Drosophilidae (Schiffer et al., 2013). Especially since it has been shown that DH_44_ play a significant role in desiccation (Cannell et al., 2016) which regulatory pathways cross-talk with cold stress pathways (Sinclair et al., 2013). This indicates that the regulation of diuresis and ion homeostasis by the nervous system and its association with cold stress resistance is a complicated process. During cold stress, the regulation of ion and water homeostasis is extremely important, as the loss of balance in both causes neuromuscular disfunction and initiates chill coma (Macmillan and Sinclair, 2011a; MacMillan et al., 2012). Therefore, future research should focus on linking changes in the levels of individual diuretic and anti-diuretic peptides with changes in water and ion homeostasis and the administration of synthetic analogs to determine whether they affect the ability to survive cold stress.

### Metabolism

At low temperature, the changes in the composition of body fluids controlled by diuresis (Neufeld and Leader, 1998; Williams and Lee, 2011) are accompanied by major metabolic changes – mainly related to carbohydrate metabolism, which is under neuroendocrine control. This is related to the production and storage of substances that are used to tolerate stress and survive low temperatures (Terhzaz et al., 2018). These substances are (a) ice nucleating agent (INA) proteins and lipoproteins in the hemolymph or cells, which induce freezing in a controlled way; (b) anti-freeze proteins (AFPs), which adsorb to the surface of small ice crystals, inhibiting its growth; and c) compatible solutes/cryoprotectants (CPAs) such as polyols and sugars (Fuller, 2004). Glycerol is the most widely occurring CPA in insects, although other polyhydric alcohols and some sugars such as trehalose have similar cryoprotective functions. These compounds, by adding to the pool of solute molecules, affect the osmotic pressure of the hemolymph and help to regulate cell volume during extracellular ice formation, and they also stabilize proteins. They are produced mainly from glycogen stored in the fat body and, in many insects, begin to accumulate at the beginning of the overwintering period (Doucet et al., 2009).

Among the different neurohormones, two highly conserved neural signaling systems have been found that are crucial for different aspects of insect metabolism and food response and are particularly involved in resistance to low temperatures (Lingo et al., 2007). These are ILPs and their receptor and NPFs, an analog of mammalian neuropeptide Y (NPY) (Lingo et al., 2007).

The tuning of insulin signaling during stress is one of the most important response of the neuroendocrine system to unfavorable conditions (Luo et al., 2012). In insects, different numbers of ILPs have been found in various species. One such peptide was found in the locusts *Locusta migratoria* and *Schistocerca gregaria*, whereas 38 in the silkmoth *Bombyx mori* (Nässel and Vanden Broeck, 2016). The classification of insect ILPs as insulin-like is mainly based on similarities in the amino acid sequences of the mature peptides to those of insulins in mammals. The number and positions of cysteine residues (Nässel and Vanden Broeck, 2016) and the arrangement of the precursor with the B, C, and A chains, which can be processed into dimeric peptides or with the maintenance of C peptides as in the insulin-like growth factor (IGF) are crucial in this assignment. Most of the research on the exact mechanisms of ILPs release and modes of action have been performed only on the model insect *Drosophila melanogaster*. It was shown that ILPs release from brain insulin producing cells (IPCs) in adult flies is triggered by a sugar meal through the direct activation of these cells via autonomous glucose-sensing capacity (Park et al., 2014). Thus, the mechanisms of glucose-induced ILPs release resemble those in pancreatic beta cells of mammals and include an ATP-sensitive potassium channel (K_*ATP*_), a glucose transporter (GluT1) and voltage-sensitive calcium channels. However, different neurotransmitters, neuropeptides and peptide hormones have been implicated in acting on IPCs to modify the expression of ILPs. These are GABA, 5-HT, OA, sNPF, TRP, corazonin, allatostatin A, CCHamide, AKHs, adiponectin, and limnostatin (Nässel and Vanden Broeck, 2016). Thus, in insects, the synthesis and release of multiple ILPs is under complex control. The system is tightly regulated and probably, as shown below fragile to unfavorable conditions such as low temperature (Li et al., 2020). This whole precisely regulated system in insects is responsible for the regulation of a number of functions, including reproduction and development, growth, metabolic homeostasis, longevity and stress response (Rauschenbach et al., 2008).

In the endocrine stress response, ILPs have been shown to play a crucial role together with biogenic amines (5-HT, OA, and DA), 20-hydroxyecdysone and juvenile hormone (JH) – hormone in adult insects, levels of which act and change similarly under stress (Gruntenko and Rauschenbach, 2018). The insulin/IGF signaling system (IIS) has been shown to respond to various stress signals such as starvation and oxidative stress. Recently, it was also suggested to play a role in temperature stress. Its participation is crucial in the regulation of the JH, OA, and DA levels, and it controls catecholamine metabolism indirectly *via* JH. Possibly one of the pathway in which the IIS is involved in the control of stress resistance is mediated through JH/DA signaling (Gruntenko and Rauschenbach, 2018). Moreover, different studies suggest the existence of a feedback loop in the interplay of JH and the IIS (Yamamoto et al., 2013). JH serves as a positive regulator of the IIS, whereas IIS negatively regulate the JH level – feedback loop (Gruntenko and Rauschenbach, 2018). Recent studies have shown that JH and DA regulate carbohydrates at the circulating carbohydrate level, mainly trehalose (used as a cryoprotectant) (Figure 1). It was shown that increases in JH and DA decrease the levels of trehalose and glucose under normal conditions but after stress exposure bring them to values close to normal. Thus, the roles of DA and JH in the neuroendocrine stress reaction in *D. melanogaster* are related to normalizing it after stress (Karpova et al., 2019). As ILPs have been shown to regulate JH and DA levels, they indirectly regulate carbohydrate levels. Moreover, Luo et al. (2012) demonstrated that IPCs in the *Drosophila* brain may be inactivated by serotonergic signaling *via* serotonin receptor 5-HT_1__*A*_. On the other hand, OA stimulates the activity of IPCs by binding to OAMB receptors, which results in an increase in cAMP and the activation of cAMP-dependent PKA (Crocker et al., 2010). Despite the knowledge about the action of biogenic amines on IPCs, we still do not know the physiological role of the antagonistic action of OA and 5-HT. As Luo et al. (2012) suggested, this action of OA and 5-HT may be associated with the tuning of insulin signaling during stress conditions.

A second signaling system that is widely known for the regulation of metabolism in insects and is connected with stress response is NPF signaling. These neurohormones were first identified in invertebrates (the tapeworm *Moniezia expansa*) based on pancreatic polypeptide antiserum (Maule et al., 1991). The first analysis showed that they are similar to mammalian NPY. In insects, an additional group of short peptides (8–10 amino acids) with similar C-terminal sequences was also discovered and named short neuropeptides F (sNPF). Recent phylogenetic analysis revealed that they are evolutionarily distinct from one another and that only long (36 amino acid) neuropeptides F are related to NPY (Fadda et al., 2019).

In insects, NPF have been involved in the regulation of different biological processes, including growth and reproduction, nociception, the circadian clock, learning, feeding and metabolism, and they act mainly as neuromodulators or neurohormones (Fadda et al., 2019). The most extensive studies of NPF functions in insects have been conducted in *D. melanogaster.* First studies of NPF physiological function were performed on their role in feeding regulation. This was based on two evidences. Firstly, earlier studies showed that NPF signaling in model nematode *C. elegans* is involved in foraging behavior and secondly NPF and its receptor (NPFR1) are similar in structure to mammalian NPY and NPY receptor (NPYR) in whom they have been known to regulate feeding (Nässel and Wegener, 2011). In studies conducted on *D. melanogaster* it was shown that NPFR is connected with transient receptor potential channel (TRP) – *painless* (pain) when respond to noxius stimuli or various stress conditions (Rosenzweig et al., 2008; Xu et al., 2008). This TRP channels are crucial for the response of flies to temperature, mechanosensory stimuli or noxious chemicals (Tracey et al., 2003). The receptor is activated by fructose so it can trigger behavior which is related to food aversion. It was shown that it is inactivated by NPF during feeding when larva reside in environment very rich in sugar (Nässel and Wegener, 2011). Thus, NPF signaling is crucial for metabolism and food acquisition. During exposure to low temperature (11°C for 120 min), the overexpression of the NPF receptor in *D. melanogaster* was sufficient to trigger cold-resistant feeding activity normally associated with fasted larvae (Lingo et al., 2007). This is evidence that during exposure to low temperature, NPF signaling may be responsible for food acquisition to store carbohydrates, which will work as cryoprotectants.

Among the 32 NPF families in insects, there are many neurohormones that might also be responsible for the regulation of metabolism during various unfavorable conditions, including cold. These neurohormones may include tachykinins and/or AKHs. However, the number of studies on the neuropeptidergic regulation of metabolic adaptations to low temperature is rather limited. AKHs have been shown to regulate, together with JH, the ice nucleator level (mainly lipoprotein) in the hemolymph of the stag beetle *Ceruchus piceus* (Xu et al., 1990) (Figure 1). Two hours after injection of AKH the level of ice nucleator increased and this increase appeared to be the result of the release of lipoproteins from the fat body (Xu et al., 1990). This is probable especially when we consider that the major function of AKH in insects is to regulate the lipids, carbohydrates and amino acid metabolism (Gäde, 2009). This might be especially important during cold. Xu et al. (1990) showed also that in lipoprotein release JH is also involved. This hormone decreased the ice nucleator activity but increased its level (Xu et al., 1990). However, the released lipoprotein pool was inactive in *Ceruchus piceus* beetle. This is in line with all the other mechanisms described above showing the crucial role of JH in the endocrine stress response.

Very recently the first detailed study about neuroendocrine stress response has been released. It describes changes in neuropeptide and neuropeptide receptors expression in *Bemisia tabaci* (Li et al., 2020). The authors showed that when insects were exposed to low temperature (4°C) for 1 and 4 h the expression level of several neuropeptides genes and neuropeptides receptor genes have been changed – for details see Table 1. These include neurohormones known for diuresis regulation, metabolic peptides and peptides which regulate reproduction and development. Remarkably peptides so far known only for myotropic properties such as proctolin or myosupressin were also changed.

**TABLE 1:** Changes in mRNA level of insect neuropeptides after short (≤1 h) and prolonged (≥4 h) cold exposure.

**Neuropeptide**	**Short cold stress**	**Prolonged cold stress**
Adipokinetic hormone	n.e.	↑
Allatostatin A (FGL/AST)	↑	↓
Allatostatin CCC	↑	↑
Capability peptide	↑	↓/↑**
CCHamide	↓	n.e.
CNMamide	↓	↑
Corazonine	↓	n.e.
Eclosion hormone	n.e.	↓
Insulie-like peptide	n.e.	↑
Ion transport peptide	n.e./↑	↓
Kinin	n.e.	n.e./↑*
Myosuppressin	n.e.	↓
Orcokinin	n.e.	↑/↓
Proctolin	n.e.	↓
RYamide	↑	↓

Prepared based on the Li et al. (2020), Terhzaz et al. (2015), and Terhzaz et al. (2018). *The effect was observable during recovery time after cold stress in D. melanogaster and no effect was observed in B. tabaci during cold stress. **Li et al. (2020) showed decrease in capa level in B. tabaci after 4 h of cold stress, whereas Terhzaz et al. (2015) a significant increase in D. melanogaster after 6 and 24 h of cold stress, the arrows represent an increase (↑) and a decrease (↓) in gene expression. n.e., no effect observed.

## Conclusion and Perspectives

In this review, we summarize the current knowledge about the neuroendocrine stress response to low temperature. Remarkably, despite the recent advance in insect neuroendocrinology, very little is known about the neurohormonal regulation of this process. Of course, some universal mechanisms typical of physiological adaptations to various unfavorable conditions are known, and only a few studies focus on cold stress.

To date, it has been shown that three major groups of compounds are involved in the response to temperature stress: biogenic amines (5-HT, OA, DA), gonadotropins (JH, 20E) and neuropeptides (ILPs, CAPA, kinins). The adjustments during exposure to low temperature include changes in overall metabolism, mainly the production and storage of cryoprotectants and loss of ion and water homeostasis due to a switch in MT and/or gut physiology. These two processes seem to be independent. First, the central role might be played by IPCs in the brain, that release ILPs which regulate DA metabolism *via* JH. This model was proven at least for high-temperature stress (Gruntenko and Rauschenbach, 2018). It should also be evaluated for low-temperature stress. IPCs are also under strict neuroendocrine control from other neuropeptides, such as sNPF, TRPs, corazonin, and AKH. Taken together, the single reports on the influence of neuropeptides on different physiological processes under stress conditions, indicate that the involvement of these peptides in neuroendocrine cold stress response should be evaluated. The roles of certain peptides could be multiple, such as the regulation of ILPs release and the regulation of the functioning of other processes, for instance, in the fat body.

On the other hand, large scale analysis of neuropeptidome showed that neuroendocrine response to cold might be complex and involve several neuropeptides, at least on mRNA level. So far only *Drosophila* has been studied, so further analysis should be performed in non-model species including bigger insects such as cockroach *Gromphadorhina coquereliana* or beetles *Tenebrio molitor* and *Nicrophorus vespilloides*. These responses might be also species specific.

Second, the exact role of biogenic amines under cold stress should also be studied. In the response to heat stress, they were shown to be intermediary in the interplay between JH and 20E, and DA metabolism is regulated by ILPs indirectly by JH. However, the other properties of catecholamines indicate that they might play pleiotropic roles in the cold response, also regulating the level of neuropeptides. Finally, the exact signaling mechanism of fluid secretion during exposure to low temperature and the involvement of all neurohormones in this process should be evaluated in detail. To date, CAPA neuropeptides and kinins have been shown to be involved. However, no neuroendocrine-controlling mechanism has been proposed thus far.

## Author Contributions

JL, AU, and PM were responsible for the general idea of the manuscript and text editing. JL coordinated the Introduction and Diuresis sections. AU coordinated the biogenic amine description. PM coordinated the Metabolism and Conclusion sections. JL, AU, PM, HC, and H-JP were responsible for the review and writing of the first draft of the manuscript. JL and AU acquired funding.

## Conflict of Interest

AU was employed by company HiProMine S.A. The remaining authors declare that the research was conducted in the absence of any commercial or financial relationships that could be construed as a potential conflict of interest.
